# Early Referral to an ALS Center Reduces Several Months the Diagnostic Delay: A Multicenter-Based Study

**DOI:** 10.3389/fneur.2020.604922

**Published:** 2020-12-18

**Authors:** Marina Martínez-Molina, Herminia Argente-Escrig, Margarita F. Polo, David Hervás, Marina Frasquet, Victoria Cortés, Teresa Sevilla, Juan F. Vázquez-Costa

**Affiliations:** ^1^Neuromuscular Research Unit, Instituto de Investigación Sanitaria La Fe, Valencia, Spain; ^2^Amyotrophic Lateral Sclerosis (ALS) Unit, Department of Neurology, Hospital Universitario y Politécnico La Fe, Valencia, Spain; ^3^Biostatistics Unit, Instituto de Investigación Sanitaria La Fe, Valencia, Spain; ^4^Department of Neurophysiology, Hospital Universitario y Politécnico La Fe, Valencia, Spain; ^5^Centro de Investigación Biomédica en Red de Enfermedades Raras, Madrid, Spain; ^6^Department of Medicina, University of Valencia, Valencia, Spain

**Keywords:** amyotrophic lateral sclerosis, diagnostic delay, diagnostic pathway, ALS Unit, diagnostic timelines

## Abstract

**Objective:** To analyze those factors contributing to the diagnostic delay in ALS.

**Methods:** Consecutive ALS patients were categorized as those studied in departmental hospitals and those studied in a referral ALS center. Demographic and clinical variables, together with data of the diagnostic pathway were collected. Multivariable models were used to assess their effect in the time between symptoms onset and the first neurologist visit (time symptoms-neurologist), in the time between the first neurologist visit and the diagnosis (time neurologist-diagnosis) and in the diagnostic delay.

**Results:** 166 ALS patients with a median diagnostic delay of 11.53 months (IQR: 6.68, 15.23) were included. The median diagnostic delay was 8.57 months (5.16, 11.61) in the referral center vs. 12.08 months (6.87, 16.8) in departmental centers. Bulbar onset, fast progression rate, upper motor neuron predominant phenotype and an early referral to the neurologist were associated with a shorter time between symptoms–neurologist. Being studied in a referral center was associated with a shorter time between neurologist–diagnosis. Comorbidities, familial ALS, bulbar onset, early referral to the neurologist and being studied in a referral center were associated with a shorter diagnostic delay. For patients studied in departmental hospitals, fast progression rate was also strongly associated with a shorter time between neurologist–diagnosis and diagnostic delay.

**Conclusion:** Unmodifiable factors (comorbidities, familial ALS, bulbar onset, and progression rate) as well as modifiable factors (early referral to the neurologist and the evaluation in an ALS referral center) have an independent effect in the diagnostic delay. The universalization of ALS Units is probably the most efficient measure to reduce the diagnostic delay.

## Introduction

Amyotrophic lateral sclerosis (ALS) is a devastating neurodegenerative disease characterized by a rapidly progressing upper and lower motor neuron (UMN and LMN) impairment that usually leads to death about 3 years after the onset of symptoms. Despite this fast progression, the diagnostic delay of ALS is ~12 months (1–14), which represents about one third of the whole life expectancy of these patients. Notwithstanding the technical advances of recent years, the diagnostic delay has not been significantly reduced in the last 20 years and it is similar among different countries and health systems (1–14). This suggests that the delay may rely on disease related factors that are difficult to modify.

This diagnostic delay supposes several drawbacks for ALS patients and for the health system. Firstly, many unnecessary tests, consultations and treatments are often performed. For example, in a previous study in Spain, up to 50% of diagnostic tests were considered unnecessary (12) and, during this diagnostic process, patients suffer the uncertainty of not having a diagnosis. Secondly, the onset of riluzole treatment and of multidisciplinary care is delayed, probably limiting their beneficial effects in ALS patients. Thirdly, a late diagnosis hampers the access to social benefits, which are already slowed down several months or even years in Spain. Fourthly, it delays the inclusion of ALS patients in clinical trials, hindering the search for new, effective treatments. When these expected treatments appear, the health system should be ready, having reduced the diagnostic delay.

Several studies have analyzed the diagnostic pathway of ALS and some factors related to the diagnostic delay (such as age, site of onset, or progression rate) have been described (1–14). However, discrepancies about these factors have emerged between studies because many of them were underpowered and only some of them performed multivariable analysis (1, 5, 8, 10, 11, 13, 14). More importantly, only few of them have assessed modifiable factors, also with controversial results (8, 11, 13, 14). Consequently, no clear strategies have been developed to reduce the diagnostic delay. In a previous study (12), we analyzed in detail the diagnostic pathway of 143 ALS patients and found that, at the beginning of the disease, patients are frequently (46%) referred to other specialists before the neurologist. Moreover, ALS was frequently (35%) misdiagnosed at the first neurology visit (12). Consequently, we hypothesized that modifiable factors (e.g., the initial diagnostic workup or being studied in a referral center) could also be responsible of this delay.

Therefore, the objective of our study was to analyze which modifiable factors independently influence the diagnostic delay of ALS, in order to design appropriate tools that allow us to reduce it in the near future.

## Methods

### Study Population

Spain has a universal healthcare coverage structured in different health departments, with one hospital caring for a specific population area. In addition, some patients have a private insurance, so they can choose to be visited in a public or a private hospital. Regarding ALS, each hospital works in a self-sufficient way with its corresponding population but all of them are part of a network that differentiates between two healthcare levels: departmental centers and referral ones. The ALS Unit of La Fe Hospital acts as departmental center for its own surrounding population (a health department of about 300,000 inhabitants), but also works as the referral center for all the other public and private hospitals in the Valencian Community (5,000,000 inhabitants). Consequently, most patients in La Fe Hospital (about 85%) are referred from other centers for diagnosis, a second opinion or to participate in clinical trials.

For this study, we reviewed the medical records of all patients with an initial clinical diagnosis of motor neuron disease, visiting, for the first time, the ALS Unit of La Fe Hospital between October 2013 and December 2017. Some of these patients had been evaluated and diagnosed in other centers, some had been evaluated in other sites and were finally diagnosed at La Fe Hospital and some others had been both evaluated and diagnosed at the referral center. Exclusion criteria were: patients in whom the ALS diagnosis was not confirmed after a minimum follow up of 2 years after the initial diagnosis, including, but not restricted to: those finally diagnosed with progressive muscular atrophy or primary lateral sclerosis as defined elsewhere (15); patients presenting cognitive or behavioral impairment before the motor symptoms' onset; and patients with missing information about their diagnostic delay.

### Clinical Variables

All patients were evaluated by the same neurologist (JFVC) who prospectively recorded demographical and clinical data: age, sex, family history of ALS, referring hospital (including if it was public or private), date and site of symptoms onset (atrophy, weakness or clumsiness), the phenotype, and the date, ALSFRS-R score, and Awaji category (16) at diagnosis. For the purpose of this study, we considered three main phenotypes (17): LMN predominant ALS (LMN-ALS), which refers to patients having no or minimal/equivocal UMN signs at the time of the diagnosis (including, but not restricted to, the flail arm and flail leg phenotypes); UMN predominant ALS (UMN-ALS), which refers to patients not meeting Awaji criteria of LMN impairment at diagnosis; and classical ALS (cALS), which include all other patients. At the time of the initial diagnosis, all cALS patients met at least criteria of possible ALS according to the Awaji criteria (16), whereas most patients with the other phenotypes did not (flail arm and flail leg phenotypes are allowed to have minimal UMN signs (17) and can therefore meet Awaji criteria). Three researchers (MM, HA, MP) retrospectively reviewed medical records from other clinics to confirm previous data and to collect new variables relative to the diagnostic pathway and delay. They can be found elsewhere (12), but the most relevant for this study are: the patient's address and comorbidities; which specialist visited the patient for the first time after motor symptoms onset (first specialist); the date of the first neurologist appointment and in which center; and the date and number of visits needed by the neurologist to make the diagnosis. The following variables were calculated with the abovementioned data: the time elapsed between symptoms onset until the patient reaches the neurologist (time symptoms-neurologist); the time required by the neurologist to make the ALS diagnosis (time neurologist-diagnosis); the time from motor symptoms onset until diagnosis (diagnostic delay); and progression rate = (48-ALSFRS-R at diagnosis)/diagnostic delay. Patient's address was categorized in cities (densely populated areas), towns and suburbs (intermediate density areas) and rural areas (thinly populated areas) according to the Eurostat degree of urbanization (DEGURBA, https://ec.europa.eu/eurostat/web/nuts/local-administrative-units). For the purpose of this article, familial ALS was defined by the presence of first- or second-degree relatives with ALS. Comorbidities were considered all those pre-existent pathologies that could mask or whose symptoms could be mistaken with ALS during the diagnostic process. It is a wide concept that includes other pathologies that impair mobility, cause dysarthria, etc… According to the center of study, patients were categorized in private vs. public health care system and in referral (La Fe Hospital) vs. departmental center (all other). Not all variables were available in all patients. The whole data collection process was supervised by JFVC, who is responsible for the veracity of the data.

## Statistical Analysis

Clinical data were summarized by mean (SD) and median (1st, 3rd Q.) in the case of continuous variables and by relative and absolute frequencies in the case of categorical variables. All time variables and the progression rate were log transformed to obtain a normal distribution. Linear regression models were performed to study the associations between response variables and predictors. The diagnostic delay is composed by two main processes in which different factors intervene: the time symptoms-neurologist and the time neurologist-diagnosis. We first analyzed those factors which could influence the two latter times. Those factors being possibly associated, were included in the diagnostic delay model, together with other factors (such as age, sex and family history of ALS) previously associated with the diagnostic delay (1, 5, 10, 13, 14). For the response variable “time symptoms-neurologist” the number of candidate predictor variables was too large, and a preselection was made by fitting a model using the relaxed elastic net algorithm. The elastic net alpha parameter was set to 0.8 and the lambda and gamma parameters, corresponding to the degree of penalty and the degree of relaxation, respectively, were determined using 500 repetitions of 10-fold cross-validation. Age and the resulting preselected variables were included in the final multivariate linear regression model. Based on the plots of the time neurologist-diagnosis and diagnostic delay, we hypothesized that the experience of the center could have a differential effect on those times, according to progression rate (see results). Namely, that rapidly progressing patients are diagnosed fast in both centers but slowly progressing patients are diagnosed faster in more experienced ones. To analyze that, an interaction between these variables was introduced in both models. Given the values distribution of the variable “progression rate” and for the purpose of the multivariable analysis, this variable was logarithmically transformed. For this reason, the progression rate values in the models and graphics are provided as logarithmic values. All analyses were performed using software R (version 3.6.3). *P*-values lower than 0.05 were considered statistically significant.

## Results

### Patients' Characteristics

**Figure 1:**
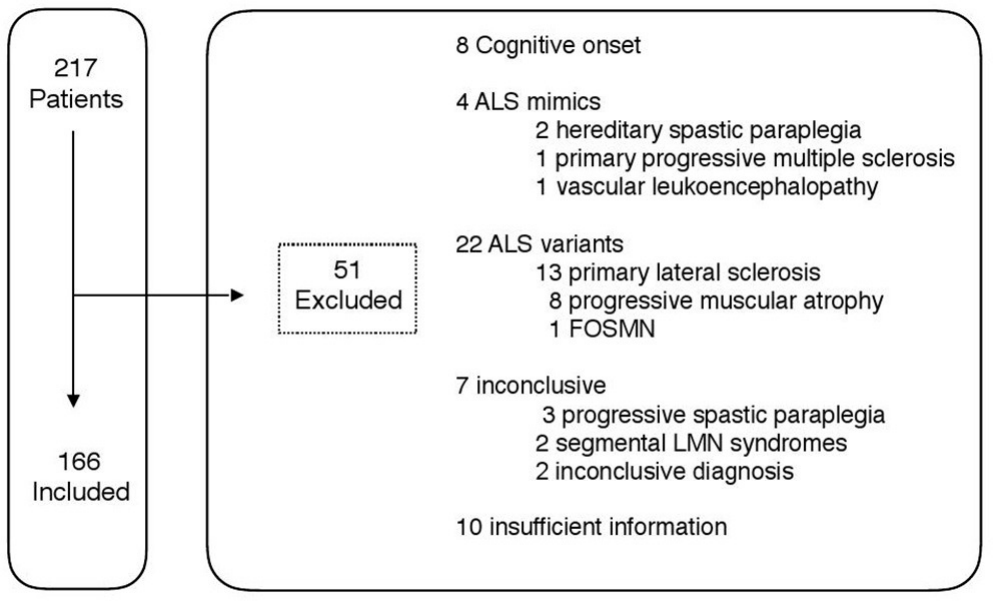
Study profile.

A total of 217 patients with an initial diagnosis of motor neuron disease, were visited for the first time at the ALS Unit of La Fe Hospital between October 2013 and December 2017. Fifty-one patients were excluded based on the abovementioned criteria (Figure 1) and 166 ALS patients were finally included in the study. Their demographic and clinical characteristics can be found in a Supplementary Table. Briefly, the median age of onset was 62 years old (IQ range: 54.47, 69.76) and there was a slight male predominance (54%). Most patients (68%) had a spinal-onset of symptoms and a cALS phenotype (70%), and 25% of ALS patients did not fulfill Awaji criteria at the initial diagnosis. A family history of ALS was present in only 16 patients (10%). Most patients (85%) had been studied in public hospitals and in departmental hospitals (83%). The median diagnostic delay was 11.53 months (6.68, 15.23). Patients needed a median of 6.45 months (3.68, 10.56) to be visited by the neurologist since symptoms' onset and the neurologist spent a median of 2.5 months (0.78, 6.82) to make the diagnosis.

**Figure 2:**
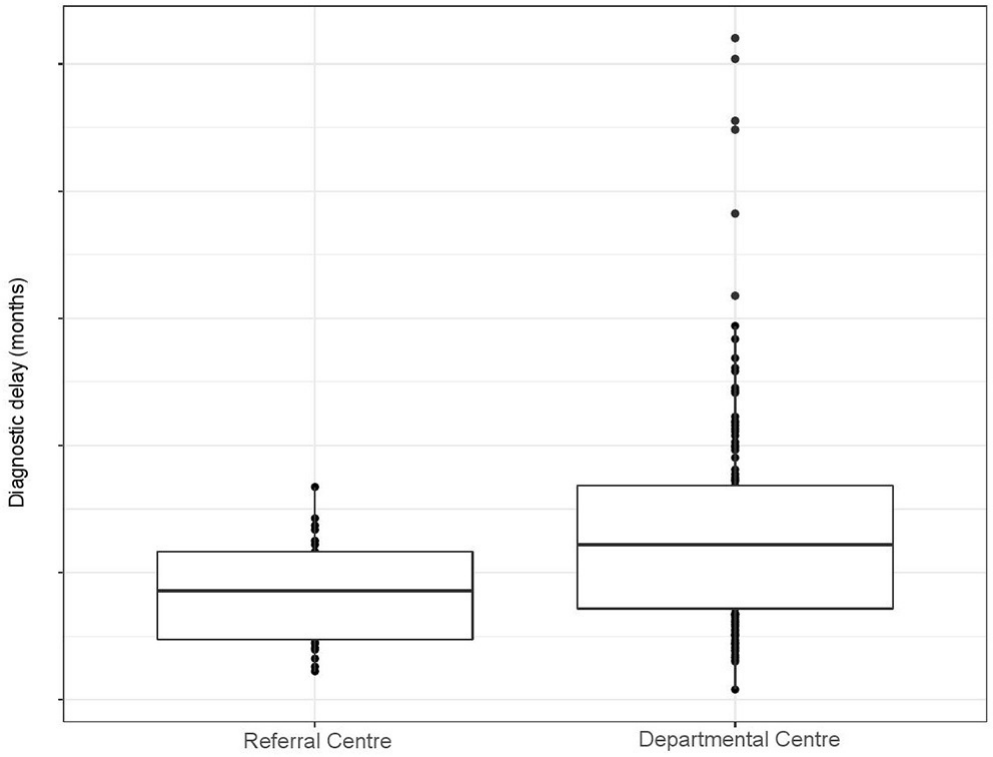
Boxplot representing the diagnostic delay in the referral and departmental centers.

Table 1 displays the characteristics of patients studied in departmental vs. referral centers. The former were older and progressed faster, but other clinical characteristics were similar. Both the time onset-neurologist and the time neurologist-diagnosis were shorter in referral centers. Despite this, the neurologist was the first specialist in about 55% of patients in both referral and departmental centers. Conversely, the neurologist needed more visits and repeated more frequently EMGs in departmental hospitals, which could explain the increase in the time neurologist-diagnosis. Accordingly, more patients studied in the referral hospital did not meet Awaji criteria at the initial diagnosis (35%) than those studied at a departmental (23%). Overall the diagnostic delay was almost 4 months shorter in the referral hospital. Figure 2 shows that, while in the referral center most cases cluster between 3 and 15 months of diagnostic delay, in the departmental center cases are widely distributed between 3 and 50 months. This suggests that both centers are able to make a fast diagnosis, but unexperienced neurologists have difficulties diagnosing some patients. Given the inequalities found in the progression rate of patients studied in each center, we hypothesized that this variable could be determining the difficulty to make the diagnosis. Namely, that the diagnosis is quite straight forward in fast progressing patients independently of the experience, but slow progressing patients are more rapidly diagnosed in referral centers (see below).

**Table 1:** Patients La Fe vs. others.

		**Referral center**	**Departmental center**
		**Mean (SD)/*n* (%) Median (1st, 3rd Q.)**	**Mean (SD)/*n* (%) Median (1st, 3rd Q.)**
Age of onset (years)		68.55 (12.54)	60.26 (11.13)
Comorbidities (no. of patients)		9 (32.14%)	33 (24.63%)
Familial ALS (no. of patients)		2 (7.14%)	14 (10.14%)
Bulbar onset (no. of patients)		8 (28.57%)	43 (32.61%)
Phenotype (no. of patients)	cALS LMN-ALS UMN-ALS	19 (67.86%)7 (25%)2 (7.14%)	97 (70.8%) 27 (19.71%) 16 (9.49%)
Awaji criteria (no. of patients)	Definite Probable Possible Not meeting criteria	4 (14.29%)6 (21.43%)8 (28.57%)10 (35.71%)	14 (10.22%) 38 (27.74%) 53 (38.69%) 32 (23.36%)
Progression rate		1 (0.82, 1.59)	0.71 (0.41, 1.16)
Diagnostic delay (months)		8.57 (5.16, 11.61)	12.08 (6.87, 16.8)
Time onset-neurologist (months)		5.05 (3.71, 9.55)	6.62 (3.68, 11.19)
First specialist neurologist (no. of patients)		15 (53.57%)	76 (55.88%)
Time neurologist-diagnosis (months)		1.27 (0.32, 2.95)	3.08 (1.08, 7.72)
Visits to neurologist (no.)		2.16 (0.9)	3.29 (2.23)
Patients with several EMG (no. of patients)		4 (14.29%)	75 (54.35%)

*Results of quantitative variables are expressed as mean (SD) for those with a normal distribution and median (IQR) for the others*.

### Time to Arrive to the Neurologist (Time Symptoms-Neurologist)

We hypothesized that several variables dependent of the patient (age, sex, years of education, degree of urbanization, comorbidities), of the health system (private vs. public hospital, first specialist visited) or of the disease (site of onset, phenotype, progression rate, familial ALS) could influence the delay to see a neurologist. All these variables were fitted to an elastic net model and six of them were preselected by the model as candidates (degree of urbanization, first specialist visited, comorbidities, site of onset, phenotype and progression rate). Age and sex were added to the final linear regression model, based on previous reports in the literature of its influence in the diagnostic delay (1, 5, 10, 13, 14). This model found that spinal onset independently associates to larger time to arrive to the neurologist, whereas UMN-ALS, higher progression rate and first referral to a neurologist shortened this time (Table 2). Conversely, age, the degree of urbanization and comorbidities showed no independent effect.

**Table 2:** Multivariable model assessing the effect of several variables in the time symptoms-neurologist.

	**Estimate**	**Std. error**	**Lower 95**	**Upper 95**	***P*-value**
Age of onset	0.005	0.005	−0.004	0.014	0.286
Towns and suburbs	0.202	0.218	−0.229	0.632	0.356
Cities	0.118	0.205	−0.287	0.523	0.565
Comorbidities	0.069	0.121	−0.169	0.308	0.566
**Spinal onset**	**0.336**	**0.12**	**0.099**	**0.573**	**0.006**
**Log (progression rate)**	**−0.448**	**0.068**	**−0.582**	**–0.314**	** <0.001**
LMN-ALS	−0.22	0.138	−0.493	0.052	0.112
**UMN-ALS**	**−0.393**	**0.181**	**−0.75**	**–0.036**	**0.031**
Private center	−0.198	0.162	−0.519	0.123	0.224
**First specialist neurologist**	**−0.364**	**0.102**	**−0.564**	**−0.163**	** <0.001**

*Bold values refers to those variables with a significant p-value (p < 0.05)*.

### Time Required by the Neurologist to Make an ALS Diagnosis (Time Neurologist-Diagnosis)

**Figure 3:**
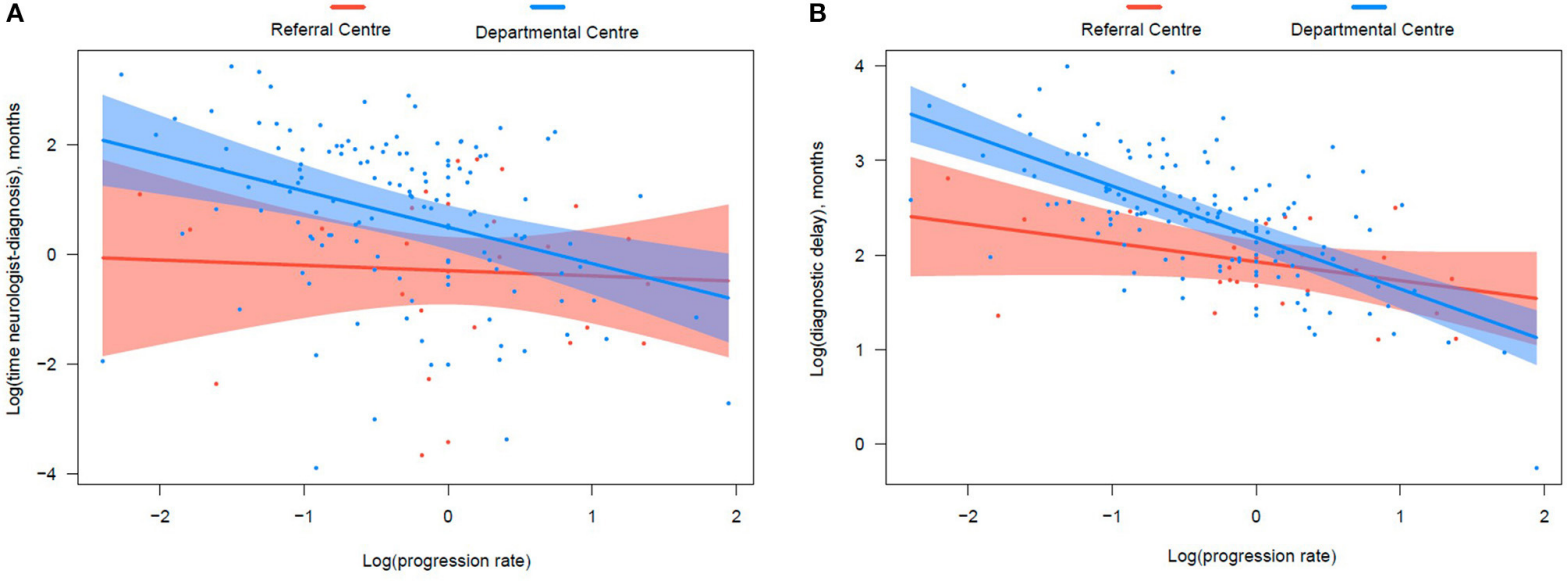
Graphical representation of the effect of the interaction between the progression rate and the center in the time neurologist-diagnosis **(A)** and diagnostic delay **(B)**. In the referral center (red), both the time neurologist-diagnosis and diagnostic delay remain relatively stable despite variations in the progression rate. Conversely, in the departmental center (blue), the progression rate has a huge influence in both times. The confidence intervals (shaded colors) are wider in graphic A than B, which explains that this effect, although visible in both graphics, is only statistically significant in the latter but not in the former model.

We also hypothesized that several variables dependent of the patient (age and comorbidities), of the health system (experience of the center) or of the disease (site of onset, phenotype, progression rate, family history) could influence the time that the neurologist needed to make an ALS diagnosis. Moreover, we hypothesized that the progression rate would have a differential effect in this time, according to the experience of the center. Consequently, we introduced an interaction between the progression rate and the center. In the multivariable model (Table 3), the progression rate, and being studied in a referral center, associated independently to an increased time neurologist-diagnosis. Since the model included an interaction between the center and the progression rate, which was not statistically significant, the association between the progression rate and the diagnostic delay is only valid for the departmental centers, whereas the association between the referral center and the diagnostic delay is only valid for the value e^0^, i.e., for a progression rate of 1. This is represented in the Figure 3A: in the referral center (red), the time neurologist-diagnosis remains stable despite variations in the progression rate. Conversely, in the departmental center (blue), greater progression rate associates with shorter time. However, the confidence intervals are wide, which explains that the interaction does not result as statistically significant in the model.

**Table 3:** Multivariable model assessing the effect of several variables in the time neurologist-diagnosis.

	**Estimate**	**Std. error**	**Lower 95**	**Upper 95**	***P*-value**
Age of onset	−0.001	0.012	−0.024	0.022	0.909
Male sex	−0.216	0.25	−0.71	0.279	0.39
Comorbidities	0.504	0.274	−0.038	1.046	0.068
Family history	−0.119	0.4	−0.909	0.671	0.766
Spinal onset	0.006	0.277	−0.542	0.553	0.984
LMN-ALS	0.313	0.319	−0.318	0.945	0.328
UMN-ALS	0.363	0.412	−0.452	1.178	0.38
**Log (progression rate)**	**−0.66**	**0.167**	**−0.99**	**−0.33**	** <0.001**
**Referral Hospital**	**−0.791**	**0.314**	**−1.41**	**−0.171**	**0.013**
Log (progression rate): referral hospital	0.548	0.369	−0.181	1.277	0.14

*Bold values refers to those variables with a significant p-value (p < 0.05)*.

### Diagnostic Delay

For the diagnostic delay model, following variables were selected based on the results of previous models: comorbidities, first specialist, site of onset, progression rate, and experience of the center. Age, sex and familial ALS were added based on previous literature (1, 5, 10, 13, 14). According to the model, familial ALS, the first specialist being the neurologist, and the referral center shorten the diagnostic delay, while comorbidities and spinal onset increase it (Table 4). Moreover, the interaction between progression rate and the center was statistically significant, highlighting a differential effect of the progression rate in the diagnostic delay: it increases the diagnostic delay much more in departmental centers than in referral ones (Figure 3B). According to the Figure 3B, patients with a progression rate of about 1 or higher (e^0^ or higher) had similar diagnostic delays in both departmental and referral centers (overlapping means and confidence intervals). However, those progressing slower can suffer an additional diagnostic delay of several months if studied in an inexperienced center. For example, patients with a progression rate of e^−1^ (0.38) would suffer an additional delay of 3 months according to the Figure 3B.

**Table 4:** Multivariable model assessing the effect of several variables in the diagnostic delay.

	**Estimate**	**Std. error**	**Lower 95**	**Upper 95**	***P*-value**
Age of onset	0.004	0.004	−0.004	0.012	0.326
Male sex	0.003	0.088	−0.17	0.176	0.973
**Comorbidities**	**0.267**	**0.096**	**0.078**	**0.457**	**0.006**
**Family history**	**−0.281**	**0.141**	**−0.559**	**−0.002**	**0.048**
**Spinal onset**	**0.219**	**0.098**	**0.026**	**0.412**	**0.026**
LMN-ALS	−0.059	0.111	−0.279	0.16	0.593
UMN-ALS	−0.013	0.145	−0.299	0.274	0.929
**First specialist neurologist**	**−0.162**	**0.081**	**0.322**	**−0.003**	**0.046**
**Log (progression rate)**	**−0.544**	**0.059**	**−0.66**	**−0.429**	** <0.001**
**Referral Hospital**	**−0.257**	**0.11**	**−0.474**	**−0.039**	**0.021**
**Log(progression rate): referral hospital**	**0.345**	**0.13**	**0.089**	**0.601**	**0.009**

*Bold values refers to those variables with a significant p-value (p < 0.05)*.

## Discussion

The diagnostic delay in ALS is about 12 months, a figure that barely changes [with few exceptions (1, 10, 17)] among health systems, countries and cultures and that also seems to have remained stable in the last 20 years (1–14). Despite an historical view of the futility of reducing the diagnostic delay in neurodegenerative diseases due to the lack of effective treatments, nowadays, it is widely accepted that diagnosing these patients at early stages of the disease will be essential to find effective therapies. Moreover, the emergence of novel and effective therapies in other neurodegenerative diseases, such as spinal muscular atrophy or familial amyloid polyneuropathy, has revealed the importance of an early diagnosis and treatment onset. Accordingly, in the last years, substantial advances have been made in the management of ALS patients, including the emergence of highly specialized ALS Units (18) and biomarkers (19, 20), which could eventually help to reduce the diagnostic delay.

Given that the ALS diagnosis is clinical and highly based on the experience of the neurologist (21), we hypothesized that the diagnostic delay could be significantly reduced in a referral center. This was demonstrated in our study, where patients evaluated in an ALS referral Unit were diagnosed in a median of 8.5 months compared to the 12 months of patients studied in departmental hospitals and of previous reports. This difference was found to be independent of other unmodifiable clinical factors and referral bias. Moreover, unlike previous studies (1, 8, 10, 11, 13), our study included a 25% of ALS patients not meeting Awaji criteria at the time of the clinical diagnosis, but in whom the ALS diagnosis was confirmed after at least 2 years of follow up. These patients, who initially only show LMN or restricted UMN signs, are more difficult to diagnose, showing a median diagnostic delay of 14–15 months in previous studies (17). Consequently, in our study the diagnostic delay is not artificially reduced with the exclusion of patients not meeting Awaji criteria and we can conclude that the median diagnostic delay in a referral ALS Unit could be of about 8.5 months rather than 12 months. Interestingly, in the referral center, more patients were diagnosed before meeting Awaji criteria, suggesting that the experience could play a major role in this specific subpopulation. However, although we did not specifically assess the influence of patients not meeting Awaji criteria in the diagnostic delay, the effect of the referral center was found to be independent of the phenotype. Since patients with LMN and UMN phenotypes were those not meeting Awaji criteria, this suggest that the effect of the referral center is applicable to all ALS patients, independently of the Awaji criteria.

Many studies have previously analyzed the influence of several factors in the diagnostic delay of ALS patients (1–14), but discrepancies about these factors have emerged because many of those studies were either underpowered or did not perform a multivariable analysis. Moreover, only a few of those performing multivariable analyses have assessed modifiable factors, also with controversial results (8, 11, 13, 14), and none have studied the effect of the experience of the center in the diagnostic delay. To prove our hypothesis and to figure out other modifiable factors, we performed several multivariable analyses assessing the effect of both modifiable and unmodifiable factors in the diagnostic delay process, which includes the time symptoms-neurologist and the time neurologist-diagnosis.

### Unmodifiable Factors

#### Age

Previous studies performing multivariable analyses showed controversial results regarding the effect of age in the diagnostic delay. Some studies proposed that aged patients have longer diagnostic delays (1, 5, 13), another (13) found the opposite and others did not find an effect (8, 11). None of these studies included covariables that vary with age such as the progression rate, comorbidities, or phenotypes (22). In our study, after adjusting for these and other covariables, an independent effect of age was not found in any of the studied time frames: the time symptoms-neurologist, the time neurologist-diagnosis, nor the diagnostic delay. This suggests that the influence of age in the diagnostic delay is not an example of ageism (that is, doing fewer diagnostic efforts by the simple fact of being old), but probably the effect of confounders (such as ALS mimics and progression rate) (21).

#### Sex

Two studies have found an independent effect of male sex in shortening the diagnostic delay (10, 14), whereas others have not (1, 5, 8, 11). Methodological differences can explain these discrepancies. Again, in any of these studies, no covariables such as comorbidities, progression rate or phenotype were considered (22). Our study shows that sex has neither an independent effect in the diagnostic delay, nor in any of its subprocesses.

#### Comorbidities

Only one study addressed the impact of comorbidities in the diagnostic delay, although it focused only on neurologic comorbidities (14). That study found longer diagnostic delay in patients with neurologic comorbidities, but it was not found to be an independent predictor variable. In our study, comorbidities were associated with a longer diagnostic delay, probably due to a combination of longer time symptoms-neurologist and neurologist-diagnosis, since a non-statistically significant trend was found in both times.

#### Site of Onset

Previous studies have consistently shown longer diagnostic delay in spinal onset patients (1, 5, 10, 11, 13, 14), although they did not adjust for other potentially confounding factors such as the progression rate, comorbidities or phenotype. We found that, independently of these and other factors, spinal onset is associated with a longer time symptoms-neurologist and diagnostic delay, but not to longer time neurologist-diagnosis. Those differences were largely attributable to lower limb onset patients in previous studies (12, 13). These patients represent probably the most challenging work up for general practitioners and other specialists, given the high frequency of other diseases (disk herniation, arthrosis, polyneuropathies, etc...) causing gait disorders in elderly populations. Conversely, these diseases are usually not challenging mimics for a neurologist, which explains that it did not affect the time neurologist-diagnosis.

#### Phenotype

A previous population study found considerable differences in the diagnostic delay according to the phenotype (17) and another study found that cALS associated with reduced diagnostic delay compared with atypical phenotypes (13). In our study, the UMN phenotype was associated with shorter time symptoms-neurologist, probably because UMN symptoms and signs are more easily identified by the general practitioner as “neurological” than LMN ones. However, we did not find an independent effect of the phenotype in the time neurologist-diagnosis nor in the diagnostic delay. Probably, other variables, such as age, sex, and progression rate, which differ among phenotypes (17, 22), would explain the differences found in previous studies.

#### Progression Rate

Although the progression rate is commonly seen as a major conditioning factor in the diagnostic delay (21), it has been scarcely studied. Nzwalo et al. found that a long diagnostic delay (>45 months) is associated with a slow disease progression (10), but little is known about its effect in the usual diagnostic delay. We found that the progression rate has a huge effect in the time symptoms-neurologist, but that its effect in the time neurologist-diagnosis and in the diagnostic delay, largely depends on the experience of the center (see below).

#### Family History

Only a previous study addressed this issue, finding a reduction in the diagnostic delay in familial ALS patients (5). Our study confirms this independent association.

### Modifiable Factors

#### Sociocultural Factors

A previous study failed to find statistically significant differences in the diagnostic delay according to the educational level, the distance from the hospital or the size of the population (14). Our study confirms that sociocultural factors are not associated with the time needed to be visited by the neurologist, suggesting that there are no sociocultural barriers hindering the diagnostic pathway.

#### Health System Factors

The fact that several studies in different countries with diverse health systems found comparable diagnostic pathways and delays, suggest that factors related with the structure and covering of the health system have little impact in the diagnostic delay, at least in developed countries. A previous study (6) found a reduction in the diagnostic delay when the patient arranged a private consultation, probably due to accelerated visits. However, two previous study found that the private health insurance neither affects the time to arrive to the neurologist nor the diagnostic delay (5, 12). Similarly, the present study did not find an independent effect in the time to arrive to the neurologist (12). Interestingly, previous efforts to establish a fast-track referral system have revealed ineffective in reducing the diagnostic delay (6). This suggests that, more important than the structure, could be the knowledge or experience of the screening physicians, usually general practitioners and neurologists. Regarding the experience of the general practitioners, previous results are controversial, with some studies (2, 9, 10, 14), but not others (4, 6, 8, 11), finding an association of an early referral to a neurologist with shorter diagnostic delay. These controversial results are probably attributable to methodological differences and to the lack of adjustment by confounding factors. Moreover, that an early referral to the neurologist results in an earlier diagnosis depends also on the ability of the neurologist to make an early diagnosis, which could also explain that previous fast-track referral experiences have failed (6). Regarding the experience of the attending neurologist, a previous study found a shortening of 3 months in the diagnostic delay between patients who visited ALS Units vs. those visited veterans' affair medical centers (13). However, it was not found to be an independent predictor in the multivariable model. Moreover, veterans' centers are not common in other health systems, and consequently this comparison might not be generalizable.

Our study sheds some light on this complex matter, confirming that an early referral to a neurologist independently associates with a reduction in both, the time symptoms-neurologist and the diagnostic delay. More importantly, being studied in a referral center (vs. departmental one) resulted in a significant reduction in both the time neurologist-diagnosis and the diagnostic delay, in the latter having a greater effect than an early referral to the neurologist. This reduction, which can be of several months in the diagnostic delay, largely depends on its effect in normal or slow progressors, while fast progressors (progression rate > 1) (23) are diagnosed similarly quickly in both referral and departmental centers (Figure 3). The effect of the referral center could be due to a higher clinical experience. However, it could also rely on the availability in the referral center of some potentially useful biomarkers (ultrasonography for the detection of fasciculations (24), brain iron deposits (25) and neurofilament light chain) to support the clinical diagnosis in those atypical or more slowly progressing patients. The impact of these biomarkers in the diagnostic delay should be analyzed in further studies.

According to these results, to effectively reduce the diagnostic delay in ALS, fast-track referral systems should be accompanied by educational plans targeting general practitioners and general neurologists. This could be a successful approach in a country such as Spain, with a universal healthcare coverage and a pyramidal structure: where general practitioners, at the base of the pyramid, perform the first symptoms' screening; while general neurologists, positioned in an intermediate level of assistance, are responsible of referring patients with suspected ALS to highly specialized centers.

### Strengths and Limitations

The main strength of our study is its design with a systematic collection of variables in a cohort of well-studied patients. To the best of our knowledge, our study is the most comprehensive one, with regards to the amount of variables collected. Moreover, the statistical approach allows an independent analysis of the effect of these variables in the different processes that result in the diagnostic delay. Our sample has the particularity that is composed by two sub-cohorts (those entirely studied at the ALS Unit of La Fe Hospital and those studied at other hospitals and afterwards derived to the referral hospital). This allowed us to analyze, for the first time, the effect of the study in different centers in the diagnostic delay. Moreover, although we studied a center-based cohort, this is probably representative of the general ALS population, since over 50% of ALS patients of the Valencian Community visit our center. Furthermore, the demographic and clinical characteristics of our series are comparable to population-based cohorts (17). In addition, several covariables were introduced in the model to reassure that the differences in the diagnostic delay between centers is not attributable to referral bias (i.e., that patients referred from departmental hospitals are more difficult to diagnose than those directly studied in the referral hospital). A limitation of our study is that some ALS patients were referred from departmental centers without a diagnosis, being finally diagnosed in our hospital. However, this would not affect our conclusions, since these patients were considered to be studied in departmental hospitals. Consequently, the differences in the diagnostic delay between departmental and referral centers could be even greater if those patients would have made the whole diagnostic process in their departmental hospitals.

### Conclusion

Our study shows that unmodifiable factors (comorbidities, familial ALS, bulbar onset and progression rate) as well as modifiable factors (early referral to the neurologist and the evaluation in an ALS referral center) have an independent effect on the diagnostic delay. Moreover, the evaluation in an ALS referral center would specifically reduce the diagnostic delay in normal and slow progressors, but probably not in fast progressors. Therefore, health care and educational plans should be designed and implemented with the aim to reduce the diagnostic delay, specifically targeting general practitioners and general neurologists. However, the establishment and reinforcement of ALS Units is probably the most efficient measure to reduce the diagnostic delay, while reassuring the best care for ALS patients.

## Data Availability Statement

The raw data supporting the conclusions of this article will be made available by the authors, without undue reservation.

## Ethics Statement

The studies involving human participants were reviewed and approved by the Ethical Committee for biomedical research of La Fe Hospital. The patients/participants provided their written informed consent to participate in this study.

## Author Contributions

MM-M participated in clinical data acquisition and interpretation and drafted the manuscript. HA-E, MP, MF, and VC participated in clinical data acquisition. TS critically revised the manuscript. DH participated in the design of the study and data analysis and interpretation. JV-C designed the study, participated in clinical data acquisition and interpretation, edited the manuscript, and had full access to all of the data in the study and takes responsibility for the integrity of the data and the accuracy of the data analysis. All authors have approved the submitted version of the paper. All authors contributed to the article and approved the submitted version.

## Conflict of Interest

The authors declare that the research was conducted in the absence of any commercial or financial relationships that could be construed as a potential conflict of interest.
