# Supplementary material for: Early Referral to an ALS Center Reduces Several Months the Diagnostic Delay: A Multicenter-Based Study
Source: Front Neurol. 2020 Dec 18;11:604922. doi: 10.3389/fneur.2020.604922 (PMC7775542; doi:10.3389/fneur.2020.604922)
Supplement: Supplementary file 1 [file Table_1.DOCX]

Supplementary Material

Supplementary Table 1. Demographical and clinical characteristics of ALS patients

|  |  |  |
| --- | --- | --- |
| Age at onset (years) | median [IQ range] | 62.09 (54.47, 69.76) |
| Sex | male  female | 90 (54.22%)  76 (45.78%) |
| Years of education | Median [IQ range] | 8 (8, 12) |
| Degree of urbanisation | cities  towns  rural areas | 103 (62.8%)  50 (30.49%)  11 (6.71%) |
| Comorbidities |  | 42 (25.93%) |
| Family history of ALS |  | 16 (9.64%) |
| Site of symptoms’ onset | bulbar-onset  spinal-onset | 53 (31.29%)  113 (68.07%) |
| Phenotype at diagnosis | cALS  LMN-ALS  UMN-ALS | 116 (70.3%)  34 (20.61%)  15 (9.09%) |
| Progression rate | median [IQ range] | 0.78 (0.44, 1.22) |
| Awajii category at diagnosis | definite  probable  possible  suspicion | 18 (10.91%)  61 (36.97%)  44 (26.67%)  42 (25.45%) |
| Health system of origin | public  private | 142 (85.54%)  24 (14.46%) |
| Hospital of origin | Hospital La Fe (Referral)  Other (Departamental) | 28 (16.87%)  138 (83.13%) |
| Neurologist as the first specialist visited |  | 91 (55.49%) |
| Time to neurologist (months) | median [IQ range] | 6.45 (3.68, 10.56) |
| Time neurologist-diagnosis (months) | median [IQ range] | 2.5 (0.78, 6.82) |
| Diagnostic delay | median [IQ range] | 11.53 (6.68, 15.23) |
